# Inpatient Outcomes of Pancreatic Cancer Surgery in Patients with Coronary Artery Disease

**DOI:** 10.3390/cancers18121980

**Published:** 2026-06-18

**Authors:** Justin Baik, Faizan Khajar, Maninder Randhawa, Harshank Patel, Aritra Paul, Dylan Yu, Scott McGuire, Osama Ahmed, Austin Brubaker, Santhosh K. G. Koshy

**Affiliations:** 1 Department of Internal Medicine, Western Michigan University Homer Stryker M.D. School of Medicine, Kalamazoo, MI 49008, USA; faizan.khajar@wmed.edu (F.K.); monty.randhawa@wmed.edu (M.R.); harshank.patel@wmed.edu (H.P.); aritra.paul@wmed.edu (A.P.); dylan.yu@wmed.edu (D.Y.); scott.mcguire@wmed.edu (S.M.); osama.ahmed@wmed.edu (O.A.); santhosh.koshy@wmed.edu (S.K.G.K.); 2 Department of Biomedical Informatics, Western Michigan University Homer Stryker M.D. School of Medicine, Kalamazoo, MI 49008, USA; austin.brubaker@wmed.edu

**Keywords:** pancreatic neoplasms, coronary artery disease, pancreatic surgery, perioperative outcomes

## Abstract

Background: Coronary artery disease (CAD) is an important comorbidity that may increase perioperative cardiovascular risk in major noncardiac surgery. However, data evaluating its impact on outcomes following pancreatic cancer surgery remain limited. This study evaluated inpatient outcomes among patients undergoing pancreatic cancer resection with versus without CAD in the United States. Methods: We performed a retrospective analysis using the National Inpatient Sample (2016–2022). Adult hospitalizations with ICD-10 diagnosis codes for pancreatic cancer and procedure codes for pancreatic resection were identified and stratified by the presence of CAD. The primary outcome was in-hospital mortality. Secondary outcomes included length of stay, hospitalization cost, and complications such as shock, respiratory failure, acute kidney injury, and transfusion. Results: A total of 49,395 hospitalizations were identified, including 6910 (14.0%) with CAD. Patients with CAD were older and had a greater comorbidity burden. In-hospital mortality was similar between groups (2.32% vs. 2.34%). Most complications were comparable, although shock was more frequent in CAD patients (6.66% vs. 5.44%). Length of stay was similar, while hospitalization costs were modestly higher in the CAD cohort. Conclusions: Pre-existing CAD was not associated with increased in-hospital mortality or longer hospitalization following pancreatic cancer surgery despite a greater comorbidity burden.

## Simple Summary

Pancreatic cancer is a highly aggressive malignancy, and surgical resection remains the only potentially curative treatment for patients with localized disease. These procedures are complex and place significant physiological stress on patients, particularly those with underlying cardiovascular conditions. Coronary artery disease is common in this population and is often assumed to increase perioperative risk, yet its impact on outcomes after pancreatic cancer surgery is not well defined. In this study, the authors aimed to evaluate whether patients with coronary artery disease experience worse short-term outcomes after pancreatic cancer surgery compared to those without this condition. Using a large national database, they examined differences in mortality, complications, hospital stay, and cost. The findings suggest that patients with coronary artery disease can undergo pancreatic cancer surgery with outcomes similar to those without it. This may help guide surgical decision making and encourage more individualized risk assessment rather than excluding patients based solely on cardiac history.

## 1. Introduction

Pancreatic cancer is one of the leading causes of cancer-related mortality in the United States [1,2]. Despite representing a smaller proportion of overall cancer incidence, it is currently the third leading cause of cancer-related death due to its aggressive nature [2,3]. The overall 5-year survival rate is low, at approximately 12–13% in recent studies [4,5]. Because most cases present at advanced stages, only about 10–20% of patients are candidates for surgical resection [6,7]. However, surgical resection remains the only potentially curative treatment for patients with localized resectable disease [8,9]. Common forms of surgical resection include pancreaticoduodenectomy (Whipple procedure) for pancreatic head tumors and distal pancreatectomy for tumors of the body or tail. These procedures are highly invasive and are associated with significant physiological stress and postoperative morbidity [10,11].

Despite advances in surgical technique and postoperative care, pancreatic resection is still considered one of the most dangerous abdominal surgeries that leads to substantial postoperative morbidity [12,13]. Serious complications, including postoperative pancreatic fistula, thrombosis of vascular reconstruction, hemorrhage, infectious complications, and cardiopulmonary events, continue to contribute to prolonged hospitalization and mortality [14]. Because patients selected for pancreatic cancer surgery tend to be older and have higher risk of comorbidities, optimization of risk factors and prehabilitation has become increasingly important in improving outcomes and expanding access to potentially curative treatment [15,16,17].

Given these conditions, patients considered for pancreatic cancer surgery generally require adequate functional status to tolerate these major procedures. Coronary artery disease (CAD) is an important comorbid condition that increases perioperative cardiovascular risk in major noncardiac surgery [18,19,20]. While perioperative risk has been extensively studied in general surgical populations, data evaluating the impact of CAD on outcomes following pancreatic cancer surgery remain limited. Therefore, we aimed to study inpatient outcomes among patients undergoing pancreatic cancer surgery with versus without pre-existing CAD in the United States.

## 2. Materials and Methods

### 2.1. Study Data

The data for this study were obtained from 1 January 2016 to 31 December 2022, using the National Inpatient Sample (NIS) database, which is part of the Healthcare Cost and Utilization Project (HCUP). The NIS is the largest publicly available all-payer inpatient healthcare database designed to produce U.S. regional and national estimates of inpatient utilization, access, cost, quality, and outcomes [21]. Unweighted, it contains data from around 7 million hospital stays each year. Weighted, it estimates around 35 million hospitalizations every year nationally. Institutional Review Board (IRB) approval was not sought due to the deidentified nature and public availability of the NIS database.

### 2.2. Study Design

We conducted a retrospective observational analysis examining inpatient characteristics and outcomes among patients undergoing surgical procedures for pancreatic cancer, stratified by the presence or absence of coronary artery disease (CAD). Adult hospitalizations (≥18 years) with ICD-10 diagnosis codes for pancreatic cancer and ICD-10-PCS procedure codes for pancreatic surgical resection (resection of the pancreas [0FTG0ZZ], duodenum [0DT90ZZ], gall bladder/common bile duct [0FT40ZZ, 0FT44ZZ, 0F190ZB], or stomach [0DB60ZZ]) were included. CAD was identified using ICD-10 codes present in any diagnostic fields.

Data extracted included patient demographics, insurance type, ZIP code-based income quartile, hospital characteristics (location, teaching status, and region), and baseline comorbidities as defined by HCUP (Table 1 and Table 2).

### 2.3. Study Outcomes

The primary outcome of interest was in-hospital all-cause mortality. Secondary outcomes included hospital length of stay, total hospitalization cost, and complications such as shock, respiratory failure, acute kidney injury, and transfusion (Table 3). We further stratified mortality by demographic characteristics, socioeconomic factors, and pertinent Elixhauser comorbidities to evaluate differences across subgroups (Table 4).

### 2.4. Statistical Analysis

All statistical analyses were performed using SAS Studio version 3.82 or SAS Proprietary Software version 9.4 (SAS Institute). Survey procedures were used to account for the complex sampling design of the National Inpatient Sample (NIS). Appropriate weights, clusters, and stratum were used on the NIS data. Survey methods were utilized for statistical analysis, including the regression models performed. Any direct or indirect unreportable frequencies (any value with a frequency less than 11) were removed from the tables to follow HCUP’s guidelines.

For Table 1 and Table 2, descriptive statistics were reported as weighted percentages for categorical variables and as medians with interquartile ranges (IQRs) for continuous variables. For comparison of baseline characteristics, continuous variables were compared using a two-sample *T*-test, while categorical variables were compared using the Rao–Scott chi-square test. The corresponding *p*-values are reported in the tables. A *p*-value < 0.05 was considered statistically significant.

**Figure 1:**
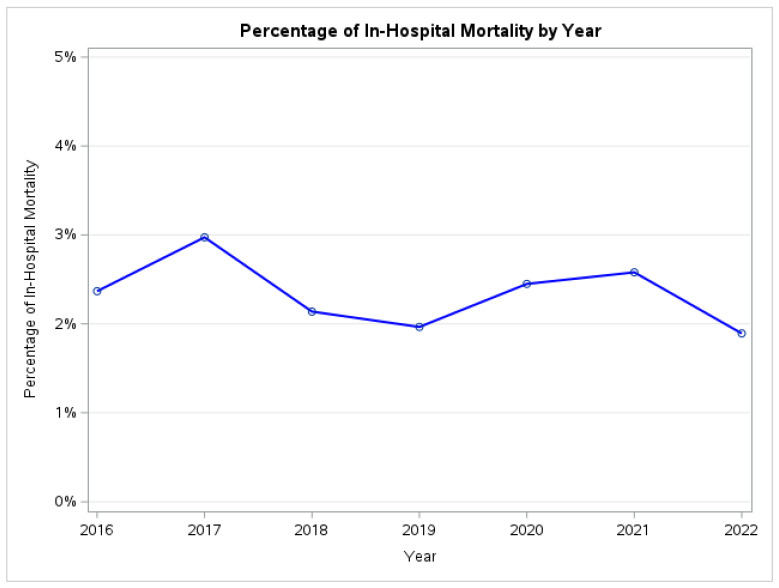
Trend of overall in-hospital mortality of pancreatic cancer patients undergoing surgery, by year in percentage.

For outcome analyses (Table 3), multivariable regression modeling was used to adjust for potential confounding. Multivariable logistic regression models were used for categorical outcomes, while multivariable linear regression models were used for continuous outcomes. Covariates included in the multivariable models were selected based on clinical relevance, with assessment for multicollinearity performed to ensure model validity. The variables kept were as follows: age, gender, race, insurance type, income quartile, urban vs. rural hospital, region of hospital, alcohol abuse, anemias due to other nutritional deficiencies, autoimmune conditions, solid tumor without malignant metastasis, cerebrovascular disease, coagulopathy, dementia, depression, diabetes without chronic complications, heart failure, mild liver disease, chronic pulmonary disease, neurological disorders affecting movement, other neurological disorders, seizures and epilepsy, obesity, psychoses, renal (kidney) failure and moderate disease, renal (kidney) failure and severe disease, hypothyroidism, peptic ulcer with bleeding, valvular disease, and weight loss. The variables removed were AIDS, chronic blood loss, leukemia, lymphoma, metastatic cancer, solid tumor without metastasis in situ, diabetes with chronic complications, drug abuse, complicated hypertension, uncomplicated hypertension, moderate to severe liver disease and failure, paralysis, pulmonary circulation disease, and other thyroid disorders. Furthermore, we adjusted the hospitalization cost to 2022 USD to account for inflation. For the subgroup analysis (Table 4), descriptive statistics were calculated, where no formal statistical testing was carried out, and the results were descriptive only. Then, a line graph was created to display the data as percentage mortality by year (Figure 1).

Throughout the analysis, adherence to the research methodological standards of the National Inpatient Sample database was maintained [22].

## 3. Results

### 3.1. Clinical Characteristics of Hospitalizations

A total of 49,395 hospitalizations for patients undergoing surgical resection for pancreatic cancer were identified, of which 6910 (14.0%) had a diagnosis of coronary artery disease (CAD) and 42,485 (86.0%) did not (Table 1). Patients with CAD were significantly older than those without CAD, with a median age of 71 years (IQR 65–76) compared with 66 years (IQR 59–73, *p* < 0.001). The CAD cohort was predominantly male (73.3% vs. 49.8%, *p* < 0.001).

White patients comprised the majority of both groups but were more prevalent among those with CAD (82.7% vs. 75.0%, *p* <0.001), whereas patients without CAD had higher proportions of Black, Hispanic, Asian or Pacific Islander, and Native American/Other patients (*p* < 0.001). Patients with CAD were more frequently insured by Medicare (72.6% vs. 53.7%), while those without CAD more often had private insurance (35.2% vs. 19.8%, *p* < 0.001).

Income distribution differed modestly between groups, with patients with CAD more commonly in the lower-income quartiles, including USD 1–55,999 (22.4% vs. 20.9%), USD 56,000–70,999 (26.0% vs. 23.6%) (*p* = 0.026). Most hospitalizations in both cohorts occurred at urban teaching hospitals (92.5% vs. 92.2%), with no significant difference in hospital location (*p* = 0.749). Regional variation was observed, with a higher proportion of CAD hospitalizations occurring in the Midwest (26.6% vs. 22.6%) and South (36.0% vs. 35.9%) compared with other regions (*p* < 0.001).

### 3.2. Comorbidities

Patients with CAD had a significantly higher burden of comorbid conditions as assessed by Elixhauser comorbidity categories (Table 2). Notably, CAD patients had a higher prevalence of diabetes with chronic complications (34.52% vs. 18.57%), heart failure (14.76% vs. 3.04%), hypertension (complicated: 23.30% vs. 7.47%; uncomplicated: 61.51% vs. 50.16%), chronic pulmonary disease (20.62% vs. 13.76%), peripheral vascular disease (10.93% vs. 5.30%), moderate renal failure and disease (11.07% vs. 4.78%), and valvular disease (9.12% vs. 3.05%) (all *p* < 0.001). Several neurologic conditions, including cerebrovascular diseases like strokes, were also more common among patients with CAD (Table 2).

On the other hand, several comorbidities, including metastatic cancer, liver disease, psychoses, and peptic ulcer disease with bleeding, were similar in both cohorts (all *p* > 0.05).

### 3.3. In-Hospital Outcomes

In-hospital mortality was higher among patients without CAD compared with those with CAD (2.34% vs. 2.32% respectively, *p* = 0.016). The incidence of shock (all forms of shock, including cardiogenic, septic, hypovolemic, other) was modestly higher in patients with CAD (6.66% vs. 5.44%, *p* = 0.019). Rates of respiratory failure, acute kidney injury, and blood transfusion were similar between groups and did not differ significantly (Table 3).

The median length of stay did not differ between patients with and without CAD (7.75 vs. 7.11 days, *p* = 0.681). However, inflation-adjusted hospitalization costs were higher among patients with CAD (USD 42,042 with IQR USD 29,947–61,061 vs. USD 40,768 with IQR USD 29,414–59,074, *p* < 0.001).

### 3.4. Mortality Trend and Sub-Analysis

Overall, in-hospital mortality of all pancreatic cancer patients with and without CAD admitted for surgery remained relatively stable between 2016 and 2022, ranging from 1.89% to 2.97%. Mortality peaked in 2017 at 2.97% and was lowest in 2022 at 1.89%. While modest year-to-year fluctuations were observed, there was a gradual decline in mortality over the study period, with a higher mortality during 2017 and then in 2020 and 2021 (Figure 1).

Mortality varied across demographic and clinical subgroups (Table 4). Patients > 65 years and males had higher mortality than younger patients and females. Racial differences were observed, with higher mortality in Black (2.43%) and Hispanic (2.66%) patients, and the lowest mortality among White patients (2.19%). Medicare insurance and lower income quartiles were also associated with higher mortality. Among comorbidities, heart failure (8.24%), peripheral vascular disease (5.51%), moderate/severe renal disease (5.29%), and alcohol abuse (4.82%) were associated with the greatest mortality.

## 4. Discussion

This national analysis of hospitalizations for pancreatic cancer patients undergoing surgical procedures demonstrates several key findings: 1. In-hospital mortality was similar, in fact, slightly lower, among patients with CAD compared to those without CAD; 2. Most in-hospital complications were comparable between groups, with the exception of shock, which was modestly higher among patients with CAD; and 3. Despite a substantially greater burden of baseline comorbidities, patients with CAD had overall short-term inpatient outcomes that were largely comparable to those without CAD.

The existing literature has consistently shown that CAD is associated with increased perioperative risk in major noncardiac surgeries [18,19,20,23,24]. Although other comorbidities such as renal failure or cerebrovascular disease are important, CAD is especially relevant due to its direct role in perioperative hemodynamic stress and ischemic risk. In contrast to these expectations, our findings demonstrate comparable, if not slightly lower, mortality among CAD patients. Several explanations may account for this observation. First, patients with known CAD may undergo more thorough preoperative cardiac risk stratification and optimization, thereby resulting in better outcomes [18,24]. Moreover, selection bias may play a role, as patients with severe or unstable CAD may be deemed poor surgical candidates and therefore not undergo operative intervention [19]. In contrast, patients with undiagnosed or subclinical cardiovascular disease will not have been risk-stratified or optimized preoperatively. As a result, the CAD cohort may paradoxically reflect a more carefully selected and medically managed population, contributing to comparable or slightly lower in-hospital mortality [18,19,24]. Additionally, despite reaching a statistically significant difference in mortality (2.32% vs. 2.34%, *p* = 0.016), the observed mortality difference between groups was only 0.02%, suggesting limited clinical significance.

This explanation may also account for the higher hospitalization costs observed among patients with CAD despite similar mortality and length of stay. As stated above, patients with known CAD have undergone much more comprehensive preoperative evaluation and perioperative management, including additional cardiac risk stratification, specialist consultation, medication adjustments, perioperative monitoring, and postoperative follow-ups. Therefore, the higher costs observed in the CAD cohort might be explained by increased efforts in perioperative optimization [25,26].

There is a structured approach to both preoperative and perioperative care in pancreatic surgery. Current evaluation extends beyond the presence of individual comorbidities and incorporates a more comprehensive assessment of nutritional status, functional capacity, and overall operative fitness [27,28,29,30]. Objective measures such as body composition parameters and functional performance, including walking speed, have emerged as important predictors of postoperative outcomes, with a faster gait speed consistently associated with improved surgical tolerance and recovery [27,30].

Complication rates were largely similar between groups, further supporting the possibility that perioperative management strategies may attenuate cardiovascular risk in CAD patients [18,24]. The observed increase in shock among CAD patients likely reflects underlying cardiovascular vulnerability [20]; however, this did not translate into higher overall mortality or longer length of stay. Notably, CAD patients demonstrated significantly higher comorbidity burden across multiple Elixhauser categories, yet inpatient outcomes remained comparable. This reinforces the fact that structured perioperative risk assessment and multidisciplinary management may offset baseline risk in this population.

A major and clinically relevant complication following pancreatic resection is the development of postoperative pancreatic fistula (POPF), which remains one of the most important and consequential adverse events after pancreatic surgery [31,32,33,34]. It has a substantial impact on postoperative recovery and surgical outcomes [33,35]. Despite advances in surgical technique and perioperative care, its incidence remains substantial, occurring in up to 41% of patients [31,33], and is associated with increased mortality in severe cases [32]. POPF is defined by the presence of amylase-rich drainage fluid resulting from failure of the anastomosis or leakage, and it typically presents after the third postoperative day [34]. Many risk factors are associated with increased fistula risk including soft pancreatic gland texture, small pancreatic duct diameter (<3 mm), obesity, increased intraoperative blood loss, younger age, high BMI, hypoalbuminemia, neuroendocrine or nonmalignant pathology, concomitant splenectomy, and vascular resection [36,37]. Due to the absence of a specific ICD-10 code, POPF could not be evaluated in our dataset. Future studies should investigate whether the incidence and severity differ between patients with and without CAD, particularly given the potential differences in tissue perfusion and healing.

Although CAD was the primary concern of interest, the subgroup findings suggest that short-term outcomes following pancreatic cancer surgery may be influenced more broadly by overall patient vulnerability in addition to cardiovascular disease in isolation. Mortality tended to be higher among groups that may reflect a higher-risk population and greater medical complexity, including older patients, male sex, black/hispanic patients, Medicare insurance status, and lower income quartiles. In particular, conditions associated with chronic end-organ dysfunction, such as heart failure and renal disease, demonstrated a stronger association with mortality than CAD itself. These findings suggest that perioperative risk in pancreatic surgery is likely multifactorial and may not be adequately captured by a single cardiovascular diagnosis alone. Instead, overall functional capacity, cumulative comorbidity burden, and the ability to tolerate the physiologic stress of major abdominal surgery may play a larger role in determining short-term outcomes.

Limitations of this study include coding inaccuracies and unmeasured confounding inherent to retrospective analyses using public databases. In addition, the National Inpatient Sample is hospitalization-based rather than patient-based, which precludes assessment at the individual patient level and limits longitudinal evaluation of outcomes. As a result, important clinical variables could not be captured, including CAD severity, functional status, prior cardiac interventions, perioperative medication use, and other patient-level factors that may influence surgical risk. The database also lacks disease- and procedure-specific information such as pancreatic cancer stage and location, laboratory values, imaging findings, operative complexity, surgical approach (open versus minimally invasive), operative duration, and estimated blood loss. Furthermore, postoperative events occurring after discharge, including readmissions, delayed complications, and longer-term mortality and survival outcomes, could not be evaluated. Finally, selection bias cannot be excluded because only patients who ultimately underwent surgical resection were included; therefore, the CAD cohort likely represents a carefully selected population considered appropriate operative candidates and may not reflect outcomes among all patients with pancreatic cancer and CAD.

## 5. Conclusions

Patients with pre-existing coronary artery disease undergoing pancreatic cancer resection did not experience higher in-hospital mortality or prolonged hospitalization compared with those without CAD, despite a substantially greater burden of baseline comorbidities. Although shock occurred more frequently among CAD patients, this did not translate into worse overall short-term inpatient outcomes. As this study only evaluates inpatient outcomes and cannot determine the broader postoperative impact of CAD, further prospective studies are needed to clarify the impact of CAD severity and long-term outcomes following pancreatic cancer surgery.

## Figures and Tables

**Table 1:** Baseline characteristics of the study population.

	Patient with CAD	Patient Without CAD	*p*-Value
Demographics			
Sample Size	6910 (14.0%)	42,485 (86.0%)	
Age (Median, IQR)	71 (65–76)	66 (59–73)	<0.0001
Gender			<0.0001
Male	5060 (73.3%)	21,135 (49.8%)	
Female	1845 (26.7%)	21,345 (50.2%)	
Race/ethnicity			<0.0001
White	5530 (82.7%)	30,910 (75.0%)	
Black	465 (7.0%)	4045 (9.8%)	
Hispanic	340 (5.1%)	3225 (7.8%)	
Asian or Pacific Islander	155 (2.3%)	1535 (3.7%)	
Native American/Other	194 (2.9%)	1515 (3.7%)	
Insurance type			<0.0001
Medicare	5020 (72.6%)	22,795 (53.7%)	
Medicaid	310 (4.5%)	3020 (7.1%)	
Private Insurance	1365 (19.8%)	14,940 (35.2%)	
Other	215 (3.1%)	1730 (4.1%)	
Income quartile			0.0262
USD 1–55,999	1530 (22.4%)	8760 (20.9%)	
USD 56,000–70,999	1775 (26.0%)	9875 (23.6%)	
USD 71,000–93,999	1620 (23.7%)	11,315 (27.0%)	
USD 94,000+	1900 (27.8%)	11,935 (28.5%)	
Urban vs. rural hospital			0.7490
Rural/Urban Nonteaching	520 (7.5%)	3295 (7.8%)	
Urban Teaching	6390 (92.5%)	39,190 (92.2%)	
Region of hospital			<0.0001
Northeast	1500 (21.7%)	8960 (21.1%)	
Midwest	1835 (26.6%)	9605 (22.6%)	
South	2485 (36.0%)	15,260 (35.9%)	
West	1090 (15.8%)	8660 (20.4%)	

**Table 2:** Baseline comorbidities of the study population.

	Patient with CAD	Patient Without CAD	*p*-Value
Elixhauser comorbidity index			
Alcohol abuse	180 (2.60%)	1065 (2.51%)	0.8287
Anemias due to other nutritional deficiencies	1280 (18.52%)	7285 (17.15%)	0.2069
Autoimmune conditions	115 (1.89%)	760 (2.10%)	0.6378
Lymphoma	65 (0.94%)	265 (0.62%)	0.1557
Metastatic cancer	2435 (35.24%)	15,285 (35.98%)	0.5932
Solid tumor without metastasis, malignant	770 (11.14%)	4050 (9.53%)	0.0569
Cerebrovascular disease	235 (3.40%)	480 (1.13%)	<0.0001
Coagulopathy	755 (10.93%)	3975 (9.36%)	0.0654
Dementia	105 (1.52%)	355 (0.84%)	0.0146
Depression	700 (10.13%)	4215 (9.92%)	0.8143
Diabetes with chronic complications	2385 (34.52%)	7890 (18.57%)	<0.0001
Diabetes without chronic complications	1305 (18.89%)	7460 (17.56%)	0.2171
Drug abuse	95 (1.37%)	525 (1.24%)	0.6663
Heart failure	1020 (14.76%)	1290 (3.04%)	<0.0001
Hypertension, complicated	1610 (23.30%)	3175 (7.47%)	<0.0001
Hypertension, uncomplicated	4250 (61.51%)	21,310 (50.16%)	<0.0001
Liver disease, mild	715 (10.35%)	4655 (10.96%)	0.5085
Liver disease and failure, moderate to severe	95 (1.37%)	745 (1.75%)	0.3085
Chronic pulmonary disease	1425 (20.62%)	5845 (13.76%)	<0.0001
Neurological disorders affecting movement	100 (1.45%)	595 (1.40%)	0.8909
Other neurological disorders	490 (7.09%)	1650 (3.88%)	<0.0001
Seizures and epilepsy	75 (1.09%)	400 (0.94%)	0.6280
Obesity	1065 (15.41%)	6085 (14.32%)	0.2877
Paralysis	95 (1.37%)	245 (0.58%)	0.0008
Peripheral vascular disease	755 (10.93%)	2250 (5.30%)	<0.0001
Psychoses	85 (1.23%)	760 (1.79%)	0.1377
Pulmonary circulation disease	210 (3.04%)	395 (0.93%)	<0.0001
Renal (kidney) failure and disease, moderate	765 (11.07%)	2030 (4.78%)	<0.0001
Renal (kidney) failure and disease, severe	125 (1.81%)	315 (0.74)	<0.0001
Hypothyroidism	895 (12.95%)	5335 (12.56%)	0.6845
Other thyroid disorders	105 (1.52%)	590 (1.39%)	0.6976
Peptic ulcer with bleeding	155 (2.24%)	1085 (2.55%)	0.4854
Valvular disease	630 (9.12%)	1295 (3.05%)	<0.0001
Weight loss	2060 (29.81%)	11,205 (26.37%)	0.0073

**Table 3:** Inpatient outcomes of the study population.

	Patient with CAD	Patient Without CAD	*p*-Value
Mortality	160 (2.32%)	995 (2.34%)	0.0156
Cost of hospitalization (2022 USD)	42,042 (29,947–61,061)	40,768 (29,414–59,074)	<0.0001
Length of hospital stay	7.75 (5.57–13.07)	7.11 (5.17–11.57)	0.6811
Shock	460 (6.66%)	2310 (5.44%)	0.0190
Respiratory failure	530 (7.67%)	2450 (5.77%)	0.2397
Acute kidney injury	1210 (17.51%)	5065 (11.92%)	0.7600
Transfusion	860 (12.45%)	4840 (11.39%)	0.5619

**Table 4:** In-hospital mortality among pancreatic cancer patients undergoing surgery, stratified by demographic and comorbidity characteristics. Values are presented as number of deaths and percentage mortality within each subgroup.

	All Pancreatic Cancer Patients Undergoing Surgery
Age	
18–65	380 (1.85%)
>65	775 (2.69%)
Gender	
Male	670 (2.56%)
Female	485 (2.09%)
Race/ethnicity	
White	800 (2.19%)
Black	110 (2.43%)
Hispanic	95 (2.66%)
Other	115 (3.38%)
Insurance type	
Medicare	745 (2.68%)
Private Insurance	310 (1.90%)
Other	100 (1.90%)
Income quartile	
USD 1–55,999	310 (3.01%)
USD 56,000–70,999	250 (2.15%)
USD 71,000–93,999	290 (2.24%)
USD 94,000+	285 (2.06%)
Hypertension, complicated/uncomplicated	685 (2.26%)
Heart Failure	190 (8.24%)
Chronic Pulmonary Disease	190 (2.61%)
Peripheral Vascular Disease	165 (5.51%)
Renal failure and disease, moderate/severe	170 (5.29%)
Alcohol abuse	60 (4.82%)
Obesity	180 (2.52%)
Diabetes (with/without chronic complication)	390 (2.05%)
Liver (mild, mod, severe)	145 (2.70%)

## Data Availability

Restrictions apply to the availability of these data. Data were obtained from the Healthcare Cost and Utilization Project (HCUP) National Inpatient Sample (NIS) database and are available from HCUP upon purchase and completion of the required Data Use Agreement. The authors are not permitted to share the data directly.

## References

[1] Bin Qadir R.M.A., Bin Umair M., Bin Tariq U., Ahmad A., Kiran W., Shahid M.H. (2024). Unraveling Pancreatic Cancer: Epidemiology, Risk Factors, and Global Trends. Cureus.

[2] Weledji E.P., Enoworock G., Mokake M., Sinju M. (2016). How Grim is Pancreatic Cancer?. Oncol. Rev..

[3] Park W., Chawla A., O’Reilly E.M. (2021). Pancreatic Cancer: A Review. JAMA.

[4] Rahib L., Coffin T., Kenner B. (2025). Factors Driving Pancreatic Cancer Survival Rates. Pancreas.

[5] Stoop T.F., Javed A.A., Oba A., Koerkamp B.G., Seufferlein T., Wilmink J.W., Besselink M.G. (2025). Pancreatic cancer. Lancet.

[6] Słodkowski M., Wroński M., Karkocha D., Kraj L., Śmigielska K., Jachnis A. (2023). Current Approaches for the Curative-Intent Surgical Treatment of Pancreatic Ductal Adenocarcinoma. Cancers.

[7] Strobel O., Neoptolemos J., Jäger D., Büchler M.W. (2019). Optimizing the outcomes of pancreatic cancer surgery. Nat. Rev. Clin. Oncol..

[8] Ryan D.P., Hong T.S., Bardeesy N. (2014). Pancreatic Adenocarcinoma. N. Engl. J. Med..

[9] Riall T.S., Lillemoe K.D. (2007). Underutilization of Surgical Resection in Patients with Localized Pancreatic Cancer. Ann. Surg..

[10] Donahue T.R., Reber H.A. (2015). Surgical management of pancreatic cancer—Pancreaticoduodenectomy. Semin. Oncol..

[11] Kolbeinsson H.M., Chandana S., Wright G.P., Chung M. (2023). Pancreatic Cancer: A Review of Current Treatment and Novel Therapies. J. Investig. Surg..

[12] Karpes J.B., Liu K., Crawford M.D., Pulitano C., Sandroussi C., Laurence J.M. (2026). Reducing Complications in Pancreaticoduodenectomy. Cancers.

[13] Caputo D., Girgis M. (2022). Editorial: Improving surgical outcomes after pancreatic resection. Front. Oncol..

[14] Henry A.C., Smits F.J., Daamen L.A., Busch O.R., Bosscha K., van Dam R.M., van Dam C.J., van Eijck C.H., Festen S., van der Harst E. (2025). Root-cause analysis of mortality after pancreatic resection in a nationwide cohort. HPB.

[15] Kim S.W. (2023). Surgical management for elderly patients with pancreatic cancer. Ann. Surg. Treat. Res..

[16] Kleeff J., Klose J., Rebelo A., Ronellenfitsch U. (2023). Editorial: Perioperative optimization of patients undergoing pancreatic surgery. Front. Oncol..

[17] Scherber P.R., Bauer J., Holländer S., Gäbelein G., Marth E., Brusokas L., Gafarli S., Jacob P., Glanemann M. (2026). Prehabilitation before pancreatoduodenectomy: Results of a retrospective single-center study. Perioper. Med..

[18] Smilowitz N.R., Berger J.S. (2020). Perioperative Cardiovascular Risk Assessment and Management for Noncardiac Surgery: A Review. JAMA.

[19] Thompson A., Fleischmann K.E., Smilowitz N.R., de las Fuentes L., Mukherjee D., Aggarwal N.R., Ahmad F.S., Allen R.B., Altin S.E., Auerbach A. (2024). 2024 AHA/ACC/ACS/ASNC/HRS/SCA/SCCT/SCMR/SVM Guideline for Perioperative Cardiovascular Management for Noncardiac Surgery: A Report of the American College of Cardiology/American Heart Association Joint Committee on Clinical Practice Guidelines. J. Am. Coll. Cardiol..

[20] Lin K., Zhou Y., Ni W., Guo K., Li Y., Ke J., Cheng L., Ni Q., Shi S., Lu Y. (2024). Assessment of perioperative cardiac risk using preoperative quantitative flow ratio in patients with coronary artery disease undergoing noncardiac surgery: A retrospective cohort study. Quant. Imaging Med. Surg..

[21] HCUP-US NIS Overview [Internet]. https://hcup-us.ahrq.gov/nisoverview.jsp.

[22] Khera R., Angraal S., Couch T., Welsh J.W., Nallamothu B.K., Girotra S., Chan P.S., Krumholz H.M. (2017). Adherence to Methodological Standards in Research Using the National Inpatient Sample. JAMA.

[23] Patel A.Y., Eagle K.A., Vaishnava P. (2015). Cardiac Risk of Noncardiac Surgery. J. Am. Coll. Cardiol..

[24] Farooq Z., Malik S., Bhat M., Farooq S. (2025). Perioperative Cardiac Complications and Evidence-Based Strategies for Their Management. Cureus.

[25] Chu H.Y., Wong C.P. (2023). Perioperative cardiac optimization. Anaesth. Intensive Care Med..

[26] Thilagar B.P., Mueller M.R., Ganesh R. (2023). Perioperative cardiac risk reduction in non cardiac surgery. Minerva Med..

[27] Kusama N., Mitobe Y., Hyodo N., Miyashita T., Baba Y., Hashimoto T., Inagaki Y. (2023). Preoperative Risk Factors in Patients with Pancreatic Cancer. J. Clin. Med. Res..

[28] Temraz S., Charafeddine M., Khalifeh M.J., Shamseddine A. (2025). Pre-Operative Markers of Post-Operative Complications in Pancreatic Cancer Patients: A Single-Center Study. J. Epidemiol. Glob. Health.

[29] Aoyama T., Katayama Y., Murakawa M., Asari M., Yamaoku K., Kanazawa A., Higuchi A., Shiozawa M., Ueno M., Morimoto M. (2015). Preoperative risk assessment of pancreatic surgery for pancreatic cancer by the surgical Apgar score. J. Clin. Oncol..

[30] Van Beijsterveld C.A.F.M., Bongers B.C., Den Dulk M., Van Kuijk S.M.J., Dejong C.H.C., Van Meeteren N.L.U. (2020). Exploring the relation between preoperative physical functioning and the impact of major complications in patients following pancreatic resection. HPB.

[31] Chui J.N., Sahni S., Samra J.S., Mittal A. (2023). Postoperative pancreatitis and pancreatic fistulae: A review of current evidence. HPB.

[32] Nebbia M., Capretti G., Nappo G., Zerbi A. (2024). Updates in the management of postoperative pancreatic fistula. Int. J. Surg..

[33] Alzelfawi L., Almajed E., AlZabin A., Alruwaili E., Alomar L., Alkhudairy A., Malaika L., AlShamrani A., Albishri S. (2024). Prevention of Postoperative Pancreatic Fistula: Systematic Review and Meta-Analysis. Surgeries.

[34] Nahm C.B., Connor S.J., Samra J.S., Mittal A. (2018). Postoperative pancreatic fistula: A review of traditional and emerging concepts. Clin. Exp. Gastroenterol..

[35] Bassi C., Marchegiani G., Dervenis C., Sarr M., Abu Hilal M., Adham M., Allen P., Andersson R., Asbun H.J., Besselink M.G. (2017). The 2016 update of the International Study Group (ISGPS) definition and grading of postoperative pancreatic fistula: 11 Years After. Surgery.

[36] Schuh F., Mihaljevic A.L., Probst P., Trudeau M.T., Müller P.C., Marchegiani G., Besselink M.G., Uzunoglu F., Izbicki J.R., Falconi M. (2023). A Simple Classification of Pancreatic Duct Size and Texture Predicts Postoperative Pancreatic Fistula: A classification of the International Study Group of Pancreatic Surgery. Ann. Surg..

[37] Ecker B.L., McMillan M.T., Allegrini V., Bassi C., Beane J.D., Beckman R.M., Behrman S.W., Dickson E.J., Callery M.P., Christein J.D. (2019). Risk Factors and Mitigation Strategies for Pancreatic Fistula After Distal Pancreatectomy: Analysis of 2026 Resections From the International, Multi-institutional Distal Pancreatectomy Study Group. Ann. Surg..

